# Quality Differences between Fresh and Dried Buckwheat Noodles Associated with Water Status and Inner Structure

**DOI:** 10.3390/foods10010187

**Published:** 2021-01-18

**Authors:** Ruibin Wang, Ming Li, Yimin Wei, Boli Guo, Margaret Brennan, Charles Stephen Brennan

**Affiliations:** 1 Institute of Food Science and Technology, CAAS/Key Laboratory of Agro-Products Processing, Ministry of Agriculture, Beijing 100193, China; ruibin.wang@lincolnuni.ac.nz (R.W.); weiyimin36@126.com (Y.W.); guoboli@caas.cn (B.G.); 2 Department of Wine, Food and Molecular Biosciences, Lincoln University, Christchurch 7647, New Zealand; Margaret.Brennan@lincoln.ac.nz

**Keywords:** extruded buckwheat flour, buckwheat noodle, LF-NMR, morphology, quality

## Abstract

Buckwheat noodles are mainly sold in the form of fresh and dried noodles in China. Among the noodles with varied proportions of extruded buckwheat flour (20% to 80%), the cooking or textural qualities of fresh and dried buckwheat noodles (FBN and DBN, respectively) were significantly different, and FBN showed a lower cooking loss and breakage ratio and were more elastic than DBN. FBN-20% showed the highest sensory score, followed by DBN-50%. The mechanisms causing the quality differences were investigated using water mobility and the internal structures of the noodles were investigated with low-field nuclear magnetic resonance and scanning electron microscopy, respectively. Compared with FBN, DBN showed a denser internal structure, which explained its higher hardness. The water within FBN and DBN was mainly in the form of softly bound water and tightly bound water, respectively. FBN with highly mobile softly bound water (longer *T*_22_) and a more uniform internal structure had a lower breakage ratio, whereas the trends of water relation with texture properties were different for FBN and DBN. The drying process and added extruded buckwheat flour together contributed to the varied cooking and textural properties.

## 1. Introduction

Buckwheat (*Fagopyrum esculentum Moench*) is widely consumed in Asian countries because of its functional components, such as rutin, and high biological value due to its well-balanced amino acid composition (rich in lysine and arginine) [1]. In China, buckwheat noodles are mainly sold in the form of fresh and dried noodles. Dried noodles are often selected owing to their easy transportation and long shelf life [2], ensuring food supply during severe emergencies.

Consumers head for buckwheat noodles with higher buckwheat contents, which requires solving the problem that the predominant proteins within buckwheat (globulin and albumin) cannot form an effective gluten network. As indicated from our previous study [3], buckwheat noodles with a high percentage of buckwheat flour (80%) can be achieved using extruded buckwheat flours. Secondly, dried buckwheat noodles with good eating qualities are favourable for commercial consumption [4]. Fresh and dried noodles are intrinsically different [5,6,7], as excessive dehydration may deteriorate the colour, texture, and even nutritional properties of the noodles [8]. However, the factors causing the differences in the eating qualities of fresh and dried buckwheat noodles (FBN and DBN) and how the reconstituted composition of buckwheat noodles influence their eating qualities are still unknown.

Moisture is one of the possible factors leading to the different eating qualities between fresh and dried noodles. During the drying process in making dried noodles, free water and softly bound water gradually migrate from the interior of the noodles to the environment, and the residual moisture is more tightly bound to the noodles [9]. As a result, a denser inner structure within the noodles is formed, which may further influence the cooking time, water absorption ratio [10], and cooking and sensory qualities of dried noodles [6,11]. Additionally, the moisture content and water status may be different in fresh and dried buckwheat noodles considering their optimum cooking time and ability to combine with water (with varying amounts of extruded buckwheat flour). Besides this, the varying amounts of extruded buckwheat flour may also generate different gluten networks and food matrixes within FBN and DBN. As reported by Drechsler and Bornhorst [12], food matrixes with different structures can bring about different textures and even nutritional properties in cereal products [8,12].

Thus, to elucidate the reasons causing the cooking and eating differences between FBN and DBN regarding water status and inner structure, low-field nuclear magnetic resonance (LF-NMR) and scanning electron microscopy (SEM) were used. A targeted improvement in the eating qualities of buckwheat noodles is expected to be achieved by understanding the mechanism causing the varying qualities.

## 2. Materials and Methods

### 2.1. Materials

Raw materials: Dehulled buckwheat was purchased from a local supermarket (Happiness Supermarket, Beijing, China) and milled (ZM 200, Retsch, Haan, Germany) by passing through it a 500 μm sieve. The buckwheat flour (dry basis) contained crude protein of 15.00%, total starch of 71.66%, fat of 2.32%, fibre of 0.37% and ash of 2.00%, respectively. Wheat flour was purchased from Jinshahe Ltd., Xingtai, China with crude protein 12.00%, total starch 85.33%, fat 1.01%, fibre 0.11%, and ash 0.61%. These chemical compositions were determined using the methods described by Wu, Tian, Liu, Li, Liang, Zhang, Liu, Wang, Zhai, and Tan [4].

Buckwheat extrusion: Buckwheat flour was mixed with distilled water to obtain a moisture content of 18.0% and equilibrated overnight at 25 °C. The buckwheat flour was extruded through a twin-screw extruder (DSE-25, Brabender OHG, Duisburg, Germany). The ratio of screw length to diameter (L/D) is 20:1, and the diameter of the die nozzle is 5 mm. The extrusion condition was under the temperature of 148 °C, screw speed of 150 rpm, and feed rate of 35 g/min [3]. The extrudates were freeze-dried using a freeze dryer (ALPHA 1-2 LD plus, CHRIST, Osterode, Germany) and milled using a grinder (ZM 200, Retsch, Germany) then through a 90-mesh sieve.

### 2.2. Noodles Preparation

Fresh buckwheat noodles: FBN were prepared following the method described by [3]. Flour (100 g, extruded buckwheat flour to wheat flour ratio of 2:8, 5:5, and 8:2) was mixed and kneaded into a dough using a dough mixer (JHMZ 200, Beijing Dongfu Jiuheng Instrument Technology Co., Ltd., Beijing, China). The dough was rested for 30 min before sheeted by a noodle-making machine (JMTD-168/140, Beijing Dongfu Jiuheng Instrument Technology Co., Ltd., Beijing, China). After sheeting, the noodle strands were cut (with a width of 2.0 mm and thickness of 1.0 mm).

Dried buckwheat noodles: FBN were dried in a humidity chamber (BCL-250-III, Beijing Luxi Technology Co., Ltd., Beijing, China) at 40 °C, 75% relative humidity for ten hours, then were further maintained at ambient conditions for 12 h for the preparation of dried buckwheat noodles.

### 2.3. Colour of Buckwheat Noodles

The colour of the uncooked DBN and FBN was measured using a Lab Colorimeter (CR400/410, KONICA MINOLTA, Tokyo, Japan). The lightness (L*), redness (a*), and yellowness (b*) were recorded as the average of 7 measurements. The total colour difference (*ΔE*) was calculated by the following Equation (1).

(1) $$\Delta E=\sqrt{{\left(L_{s}-L_{s}^{*}\right)}^{2}+{\left(a_{s}-a_{s}^{*}\right)}^{2}+{\left(b_{s}-b_{s}^{*}\right)}^{2}}$$

L_s_*, a_s_*, and b_s_* are the standard values of a white calibration ceramic plate (97.13, 0.21, and 1.87, respectively). A higher ΔE with a more considerable value indicates the colour is darker, taken as buckwheat noodles with a higher content of buckwheat flour.

### 2.4. Cooking Properties of Buckwheat Noodles

The optimal cooking time (OCT), cooking loss (CL), and cooking breakage ratio (CBR) were measured following the methods of [13] and [4]. OCT is defined as the time when the white core within the noodles disappeared with two transparent glass plates squeezing the noodle strands. CL was calculated as the number of solid substances in the cooking water at OCT. CBR was expressed as the ratio of the number of the broken noodle strands and the original number of noodle strands after cooking. All the noodles were cooked at OCT in this research.

### 2.5. Textural Properties of Buckwheat Noodles

The texture of buckwheat noodles was analysed using a texture analyser (TA-XT2i, Stable Micro Systems, London, UK). Five cooked noodle strands were placed side by side on a loading platform and were compressed by a probe (A/LKB-F) under the strain of 75%. The pre-test speed, test speed, and post-test speed were set as 2.0 mm/s, 0.8 mm/s and 2.0 mm/s, respectively. The time between two compressions was 10 s, and the trigger force was 10 g. The textural parameters were averaged from 7 measurements.

### 2.6. Sensory Properties of Buckwheat Noodles

Sensory properties of buckwheat noodles were evaluated by a sensory panel (eight members, four females and four males) according to the method of Wang, Li, Chen, Hui, Tang, and Wei [3]. The sensory panel members were carefully selected with a pre-experiment. A control noodle sample was used to test the panel’s stability. Sensory parameters and their scores were shown in Table S1.

### 2.7. Water Status and Distribution in Uncooked Buckwheat Noodles

The water status and distribution of the uncooked FBN and DBN were measured according to the method of [2] using the LF-NMR (NMI20-030H-I, Niumag Analytical Instruments Co., Ltd., Suzhou, China), which was equipped with a 0.5 T permanent magnet corresponding to a proton resonance frequency of 21 MHz at 32 °C. Transverse relaxation times (*T*_2_) was obtained from Free Induction Decay (FID) and Carr-Purcell-Meiboom-Gill (CPMG) experiments. FID sequence was used to set the system parameters using soybean oil. The number of sampling points (TD) was 10,104, the relaxation time (TR) was 1000 ms, echo time (TE) was 0.101 ms, while the number of echoes (NE) was 1000 and acquired as 64 scan repetitions. Uncooked noodle strands were put in an NMR tube with a 5 mm diameter. MultiExpInv analysis software (Suzhou Niumag Analytical Instrument Corporation, Suzhou, China) was used to fit CPMG decay curves to a multi-exponential function model.

The times of the peak position were recorded as *T*_21_, *T*_22_, and *T*_23_, which were termed as tightly bound water, softly bound water, and free water, respectively. The relative content of water in different status was calculated using the proportion of the corresponding peak area to the total area, recorded as *A*_21_, *A*_22_, and *A*_23_, respectively.

### 2.8. Scanning Electron Microscopy (SEM) Analysis

The morphology of the uncooked and cooked buckwheat noodles was characterised following the method of Wang, Li, Chen, Hui, Tang, and Wei [3]. Samples were lyophilised using a freeze-dryer (ALPHA 1-2 LD plus, CHRIST, Germany), fractured and mounted on a copper stub. Then the samples were coated using a Sputter coater (JFC-1600, JEOL, Tokyo, Japan). The morphologies of the fracture surface of noodles were observed using a scanning electron microscope (JSM-6510LV, JEOL, Tokyo, Japan), at a magnification of 40× and 500×.

### 2.9. Statistical Analysis

All the analyses were conducted in triplicate unless otherwise mentioned. ANOVA and Duncan’s Multiple Range Test were used at a significance level of *p* < 0.05, accomplished using SPSS 18.0 (IBM, New York, NY, USA).

## 3. Results

### 3.1. Cooking Properties

**Figure 1:**
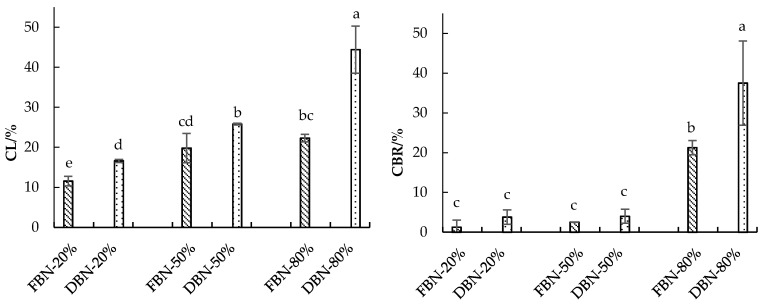
Cooking loss (CL) and cooking breakage ratio (CBR) of FBN and DBN. FBN-20/50/80% is fresh buckwheat noodles with 20/50/80% of extruded buckwheat flour; DBN-20/50/80% is dried buckwheat noodles with 20/50/80% of extruded buckwheat flour; a, b, c, and d indicate values are significantly different at *p* < 0.05.

FBN showed a shorter optimal cooking time (OCT) than DBN (Table 1), which also showed better cooking properties than DBN. For instance, the CL of DBN was higher than that of FBN (16.60–44.40% and 11.56–22.31%, respectively) (Figure 1). The cooking breakage ratio (CBR) for DBN was almost two times higher than FBN (37.5% and 21.3%, respectively) when the addition ratio of extruded buckwheat flour was 80% (Figure 1). The cooking loss (CL) of buckwheat noodles was significantly increased (*p* < 0.05) when the amount of extruded buckwheat flour was increased from 20% to 80%.

### 3.2. Textural Properties

The textural properties of DBN and FBN varied with the ratio of extruded buckwheat flour. Hardness and elasticity are the most critical texture parameters for consumers. In this study, the hardness of DBN and FBN was decreased with an increased ratio of extruded buckwheat flour (Table 1). The hardness of DBN-20% was significantly higher than that of FBN-20%, while FBN-80% showed a higher hardness than DBN-80% (*p* < 0.05). The elasticity of FBNs was significantly higher than that of DBNs. The elasticity was high and relatively stable for FBN, but a noticeable increase in the elasticity of DBN was observed with increased extruded buckwheat flour ratios (*p* < 0.05). The resilience of DBNs was also higher than FBN regardless of extruded buckwheat flour ratios. Similar results were also found regarding the cohesiveness and chewiness of DBNs and FBNs. DBN with increased proportions of extruded buckwheat flour showed increased cohesiveness while FBN showed insignificant changes. For chewiness, FBN showed a decrease when the ratios of extruded buckwheat flour increased from 20% to 80%, whereas the chewiness of DBN was the highest when the amount of extruded buckwheat flour was 50%.

### 3.3. Colour of Uncooked FBN and DBN

The colour of DBN was darker than that of FBN (Table 2), with a higher *ΔE* for DBN (values ranged from 36.56 to 58.81 of DBN, and 28.79 to 53.11 of FBN, respectively) (*p* < 0.05). DBN showed lower lightness (L*) and yellowness (b*) values than those of FBN. The L* and a* of FBN were higher than those of DBN with the same ratio of extruded buckwheat flour, whereas b* showed an opposite trend. Besides this, buckwheat noodles with 20% and 80% of extruded buckwheat flour showed significantly lower b* values than that with 50% extruded buckwheat flour (24.74 and 21.62 for FBN-50% and DBN-50% respectively).

### 3.4. Sensory Properties

The sensory evaluation reflects the direct conceiving of the consumers, which cannot be easily replaced by instrumental analysis, as some of the parameters measured by instruments cannot cover the complex feeling of humans [14]. In this study, the FBN-20% showed the highest sensory score, followed by DBN-50%, as the colour and appearance were much higher for noodles with 20% extruded buckwheat flour than for other noodles. The hardness and total scores showed a decreased trend for FBN with increased ratios of extruded buckwheat flour, while DBN showed the highest value with 50% extruded buckwheat flour. With 80% extruded buckwheat flour, the noodles tended to be soft and sticky, which showed the worst sensory quality among all noodles, with a lower score in the colour, hardness, stickiness, and smoothness.

Interestingly, there were insignificant differences observed in terms of elasticity, stickiness, smoothness, and flavour between FBN and DBN with the same ratio of extruded buckwheat flour (Table 3). Note that the TPA hardness was not increased with a higher sensory score, as the preferable hardness was given the highest score, and the preferred TPA hardness of the buckwheat noodles was approximately 167 g and 150 g.

### 3.5. Water Status and Distribution

**Figure 2:**
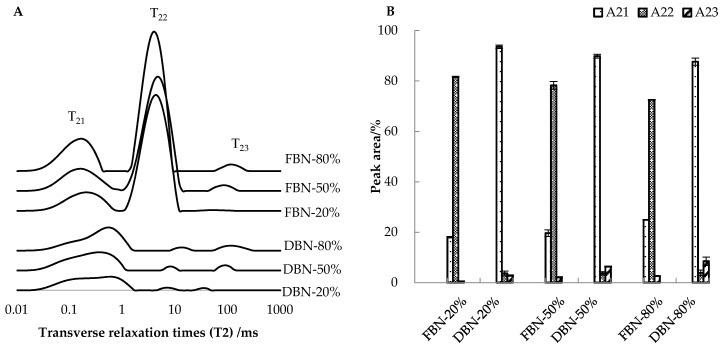
Transverse relaxation time (T2) (**A**) and the relative amount of water (A2) (**B**) in different status/distribution for uncooked FBN and DBN. FBN-20/50/80% is fresh buckwheat noodles with 20/50/80% of extruded buckwheat flour; DBN-20/50/80% is dried buckwheat noodles with 20/50/80% of extruded buckwheat flour. *T*_21_, *T*_22_, and *T*_23_ represent the transverse relaxation time of tightly bound water, softly bound water, and free water, respectively. *A*_21_, *A*_22_, and *A*_23_ are the proportion of tightly bound water (TBW), softly bound water (SBW), and free water (FW), respectively.

The water within noodles distributes differently during dough mixing and the drying process [2]. The transverse relaxation time of proton (*T*_2_) expresses the interaction between water and substances, suggesting the mobility of the water. In this study, the *T*_2_ peaks representing the water under different status appeared in three positions, i.e., about 0.16 to 0.66 ms, 4.04 to 17.34 ms, and 30.30 to 127.31 ms, respectively (Table 4, Figure 2A). The water was termed as tightly bound water (TBW), softly bound water (SBW), and free water (FW), which was similar to the results of previous studies [15,16].

For noodles with the same ratio of extruded buckwheat flour, the *T*_2_ for uncooked FBN and DBN was significantly different (Table 4) (*p* < 0.05) and the *T*_21_ and *T*_22_ of FBN were smaller than those of DBN, indicating tight bonding among water molecules (TBW and SBW) and non-water substances within FBN. However, the *T*_23_ of DBN was smaller, indicating that drying induced a stronger interaction between the free water and solids [17]. However, FBN and DBN displayed different trends with varying amounts of extruded buckwheat flour in terms of *T*_21_, *T*_22_, and *T*_23_. *T*_21_ of FBN decreased from 0.21 ms to 0.16 ms, whereas that of DBN increased with a higher addition ratio. *T*_22_ of FBN-20% was insignificant to that of FBN-50% and similar for DBN with 20% and 50% extruded buckwheat flour. However, as the amounts of extruded buckwheat flour up to 80%, FBN showed a downward *T*_22_, while DBN showed an upward *T*_22_. *T*_23_ of FBN and DBN increased with increased proportions of extruded buckwheat flour.

The water within FBN was mainly in the form of SBW, while the proportion of TBW was the highest in DBN (Figure 2B). As the main form of water, the proportion of SBW (*A*_22_) of FBN decreased from 81.60 to 72.51% (Table S2) with the increasing ratios of extruded buckwheat flour from 20% to 80%. In comparison, *A*_22_ of DBN remained unchanged (about 3.70%). The ratio of TBW (*A*_21_) of DBN decreased with increased proportions of extruded buckwheat flour (93.58–89.86–87.65%), whereas the *A*_21_ of FBN increased from 18.03% to 24.88%. *A*_23_ of DBN showed a significant increase from 2.78 to 8.50% with increased ratios of extruded buckwheat flour, higher than those of FBN. There was no simple pattern with the *T*_2_ and the *A*_2_ under a different status. The relation between the water mobility and noodle quality will be discussed in the discussion section.

### 3.6. Morphology

**Figure 3:**
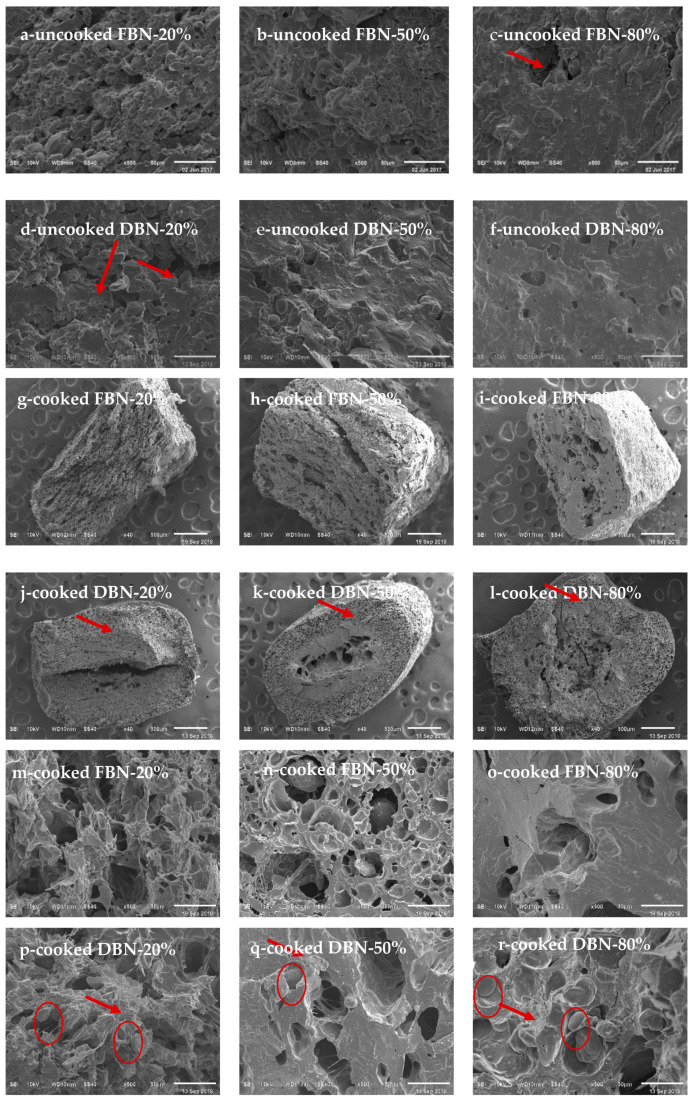
Morphology of uncooked and cooked FBN and DBN. FBN-20/50/80% is fresh buckwheat noodles with 20/50/80% of extruded buckwheat flour; DBN-20/50/80% is dried buckwheat noodles with 20/50/80% of extruded buckwheat flour. (**a**–**f**) are uncooked samples at a magnification of 500 times; (**g**–**l**) are samples at a magnification of 40 times; and (**m**–**r**) are cooked samples at a magnification of 500 times. The image resolution was 220 pixels per inch.

The morphology of fracture surfaces of uncooked and cooked FBN and DBN is shown in Figure 3. For uncooked noodles, DBN-80% (Figure 3f) showed no granular structure, where the extruded buckwheat flour formed a continuous phase similar to the protein matrix, which improved the internal network [3,18]. After drying treatment, the wheat starch granules were less exposed to the surface in DBN, and the internal structure was denser for DBN (Figure 3a–f). After cooking, the integral starch granules swelled and lost their granular structure (Figure 3m–r). The penetration of water created holes with different sizes and numbers which were finer from the outside than the inside of the cooked DBN, while the fracture surface of FBN was looser and the holes were more homogeneously distributed (Figure 3g–l). Additionally, the number of irregular holes was less for noodles with increased ratios of extruded buckwheat flour. DBN-20% and DBN-50% showed denser and less porous internal structure than the cooked FBN with the same proportion of extruded buckwheat flour (Figure 3m versus Figure 3p, Figure 3n versus Figure 3q), but the DBN-80% swelled to a higher degree, showing a looser structure, implying there was a soft texture of cooked noodles.

## 4. Discussion

Drying is the main processing procedure that is different for DBN and FBN; it changes the water status and distribution of noodles. It is well known that moisture is one of the reasons for noodles’e different eating qualities. However, the moisture content of cooked DBN and FBN were not significantly different (Table 4), although the OCT was different for DBN and FBN in this study (Table 1). On the other side, longer cooking time may result in a higher CL and CBR, as more degraded starch molecules due to extrusion may dissolve in the cooking water. Besides, processing can also induce varied water mobility and food structure, which can also affect the eating qualities. The water channels due to water migration and denser network due to moisture loss may influence the structure of the noodles [19]. For fresh buckwheat noodles with different contents of extruded buckwheat flour, the viscous extruded buckwheat flour together with the wheat gluten may create varied network structure, contributing to buckwheat noodles with a higher proportion of buckwheat flour. To verify the above inference, the correlation between the cooking properties and eating properties of buckwheat noodles with the features of water of different statuses was performed (Table 5).

### 4.1. Roles of Water Status/Distribution and Internal Structure on the Cooking Properties of FBN and DBN

CBR was correlated well with the ***T*_22_** of uncooked noodles and the moisture content after cooking (*p* < 0.05) (Table 5). *T*_22_ showed a negative correlation with the CBR of FBN, but a positive correlation with the CBR of DBN. The higher *T*_22_ of FBN was correlated with a lower CBR. A possible reason for this is that the SBW (the water that existed inside the gluten network [20]) tended to be more mobile, and highly mobile water can lead to a more uniform and developed protein network, leading to a lower breakage ratio. This uniform inner structure was confirmed by the SEM images (Figure 3a–c). In comparison, SBW was not the main form of water within DBN, and the longer *T*_22_ implied that the SBW could not be bound tightly in the structure of dried noodles (Table 4). DBN with a higher ratio of extruded buckwheat flour showed a longer *T*_22_ and a less uniform but denser internal structure, leading to a larger CBR. Thus, FBN-20% with a relatively more homogenous structure (Figure 3m) showed a higher resistance to breakage during cooking. The moisture content was negatively correlated with the CBR of FBN (Table 5), which was mainly due to the loose structure of FBN-80% (with the lowest moisture content).

The CL of FBN showed a positive relationship with the *A***_23_** (Figure 1 and Figure 2B). However, this is more likely to be due to the dissolution of the degraded amylopectin molecules [21] from extruded buckwheat flour during noodle cooking. For FBN with a higher ratio of extruded buckwheat flour, the small molecules from the extruded buckwheat flour could be released more easily into the cooking soup [22], causing the greater CL of FBN. Moreover, a weak or discontinuous protein matrix results in a protein network that is too loose and permits a large amount of extrudate to dissolve in the water during cooking [23]. DBN-80% and FBN-80% showed denser but less uniform internal structures (more holes), which explained the higher cooking loss (Figure 1 and Figure 3). Chillo, Laverse, Falcone, Protopapa, and Del Nobile [13] also found that an internal structure with more or fewer holes inhibited or improved the absorption of water, which contributed to the breakage susceptibility and cooking resistance of the noodles. However, further evidence is required to justify if it is a coincidence that the CL was correlated to the *A*_23_ or if the CL could be attributed to other parameters relating to the water status.

### 4.2. Roles of Water Status/Distribution and Internal Structure on the Textural Properties of FBN and DBN

Water mobility (Table 5) and inner structure shall be combined to explain the changes in textural properties [19]. In this study, *T*_21_ showed a positive correlation with the hardness of FBN, whereas *T*_23_ showed a negative correlation with the hardness of DBN. Both the FBN with a lower ratio of extruded buckwheat flour (longer *T*_21_) and the DBN with a lower percentage of extruded buckwheat flour (shorter *T*_23_) exhibited a more uniform but less dense protein network (Figure 3g,j,m,p), contributing to the higher hardness. The more compact internal structure led to a higher hardness, which was consistent with the study [4]. Additionally, a more continuous and denser internal structure was found for DBN-20%, with more tightly bound water and free water.

The elasticity of DBN was negatively correlated with *A*_21_ but positively correlated with *A*_23_. The reason for the changes in the elasticity of DBN and FBN is different. Firstly, the elasticity of FBN was not significantly different, and there was no correlation found between the elasticity and the parameters reflecting water status changes for FBN. Secondly, as suggested by the improvement in rubber elasticity using linear or branched polymers [24], a possible presumption is that a more degraded branched/bulk structure was maintained in DBN-20% and DBN-80%, and thus a higher proportion of free water (higher *A*_23_) was retained in the bulk structure. Meanwhile, the polymers formed from a higher content of extruded buckwheat flour may entangle with each other and/or with the proteins within the buckwheat noodles during drying, which caused increased elasticity. Without the drying treatment, FBN delivered a relatively stable elasticity (about 1.0) compared to DBN (about 0.7–1.0).

The cohesiveness of the noodles is of particular interest to us, and it can reflect the integrity of the soft food’s structure. For FBN, the cohesiveness was negatively correlated with the *T*_21__,_ *A*_21_, and *A*_22_, but positively correlated with the *T*_23_ and *A*_23_. Interestingly, a shorter *T*_21_, smaller *A*_21_ and *A*_22_, longer *T*_23_, and larger *A*_23_ were observed in FBN with a higher ratio of extruded buckwheat flour. The higher addition of extruded buckwheat flour could stick different components, including starch granules and protein, together, as indicated previously [3], leading to an improved internal structure. Besides this, the moisture content was negatively correlated with the resilience of cooked DBN (Table 5), which explained the lower resilience of DBN-80% (with a higher moisture content) after cooking.

From this study, the water distribution/status cannot solely explain the changes in the cooking or eating properties without understanding the changes in the inner structure of noodles. The relationship between the water and the texture extensively relied on the specific food system, as the form of water taking the dominant proportion might be different. Since the water distribution/status correlated well with the texture of seafood [25], if a similar relationship can be found between the water distribution/status and the internal structure, then the texture of other starch-based foods shall be further confirmed. Besides this, a joint modification of water mobility and inner structure shall be considered for the improvement of food qualities. The water absorption ability of extruded buckwheat flour and gluten still merits further investigation to help explain the water mobility within the buckwheat noodle system.

## 5. Conclusions

The cooking and eating differences between FBNs and DBNs are reflected differently in their water status and inner structure. FBN exhibited more desirable cooking qualities (lower cooking loss and breakage ratio) and a more elastic texture than DBN. The highly mobile softly bound water of FBN helps gluten network formation and results in a lower breakage ratio. The denser inner structure of DBN results in a higher hardness, and the increased cooking loss of DBN was mainly due to the smaller molecules from the extruded buckwheat flour and the less uniform and looser internal structure of the noodles. The elasticity is more likely to be attributed to the bulky structure or entanglement with the branched polymers.

## Figures and Tables

**Table 1:** Optimum cooking time and textural properties of cooked FBN and DBN.

Noodles	OCT/s	Hardness/g	Elasticity	Cohesiveness	Chewiness	Resilience
FBN-20%	140	167.01 ± 16.22 ^b^	0.99 ± 0.02 ^a^	0.61 ± 0.01 ^e^	100.28 ± 8.94 ^d^	0.46 ± 0.02 ^c^
DBN-20%	320	192.60 ± 4.05 ^a^	0.69 ± 0.12 ^c^	0.99 ± 0.03 ^c^	139.4 ± 15.90 ^b^	0.62 ± 0.02 ^b^
FBN-50%	180	145.42 ± 10.84 ^c^	0.98 ± 0.02 ^a^	0.62 ± 0.01 ^d,e^	88.79 ± 7.35 ^d,e^	0.47 ± 0.01 ^c^
DBN-50%	320	150.68 ± 15.98 ^c^	0.88 ± 0.04 ^b^	1.18 ± 0.04 ^b^	156.35 ± 14.07 ^a^	0.67 ± 0.01 ^a^
FBN-80%	160	120.08 ± 11.04 ^d^	1.03 ± 0.05 ^a^	0.64 ± 0.01 ^d^	79.10 ± 7.92 ^e^	0.42 ± 0.03 ^c^
DBN-80%	330	101.05 ± 8.85 ^e^	0.97 ± 0.02 ^a^	1.28 ± 0.02 ^a^	125.35 ± 14.53 ^c^	0.61 ± 0.01 ^b^

FBN-20/50/80% is fresh buckwheat noodles with 20/50/80% of extruded buckwheat flour; DBN-20/50/80% is dried buckwheat noodles with 20/50/80% of extruded buckwheat flour. ^a^, ^b^, ^c^, ^d^, and ^e^ in a column indicate values are significantly different at *p* < 0.05.

**Table 2:** Colour of uncooked FBN and DBN.

Noodles	L*	a*	b*	*ΔE*
FBN-20%	76.52 ± 0.46 ^a^	−1.68 ± 0.08 ^e^	21.87 ± 0.94 ^b^	28.79 ± 0.90 ^f^
DBN-20%	65.93 ± 0.91 ^b^	3.72 ± 0.08 ^c^	20.59 ± 0.52 ^c^	36.56 ± 0.76 ^e^
FBN-50%	62.75 ± 0.56 ^c^	1.83 ± 0.26 ^d^	24.74 ± 0.72 ^a^	41.32 ± 0.79 ^d^
DBN-50%	51.65 ± 1.70 ^d^	5.96 ± 0.31 ^b^	21.62 ± 0.22 ^b^	49.92 ± 1.55 ^c^
FBN-80%	48.80 ± 0.24 ^e^	7.22 ± 0.17 ^a^	22.75 ± 0.26 ^b^	53.11 ± 0.31 ^b^
DBN-80%	41.83 ± 0.72 ^f^	7.21 ± 0.18 ^a^	20.61 ± 0.57 ^c^	58.81 ± 0.54 ^a^

FBN-20/50/80% is fresh buckwheat noodles with 20/50/80% of extruded buckwheat flour; DBN-20/50/80% is dried buckwheat noodles with 20/50/80% of extruded buckwheat flour. ^a^, ^b^, ^c^, ^d^, ^e^, and ^f^ in a column indicate values are significantly different at *p* < 0.05.

**Table 3:** Sensory properties of cooked FBN and DBN.

Noodles	Colour	Appearance	Hardness	Elasticity	Stickiness	Smoothness	Flavour	Total Score
FBN-20%	6.67 ± 1.37 ^a^	7.67 ± 0.82 ^a^	17.50 ± 0.84 ^a^	18.50 ± 2.88 ^a^	15.67 ± 2.58 ^a^	7.83 ± 1.33 ^a^	3.17 ± 0.41 ^a^	77.00 ± 8.07 ^a^
DBN-20%	5.02 ± 1.05 ^a^	6.68 ± 1.32 ^a,b^	13.43 ± 1.48 ^b^	15.56 ± 1.26 ^a^	13.45 ± 3.55 ^a^	6.88 ± 1.52 ^a^	4.07 ± 0.48 ^a^	62.07 ± 7.22 ^b^
FBN-50%	3.50 ± 1.87 ^b^	5.33 ± 1.03 ^b,c^	13.33 ± 1.97 ^b^	15.33 ± 5.57 ^a^	15.17 ± 2.14 ^a^	6.67 ± 1.63 ^a^	3.50 ± 1.22 ^a^	62.83 ± 9.97 ^b^
DBN-50%	3.22 ± 1.54 ^b^	6.26 ± 1.12 ^b^	16.94 ± 1.07 ^a^	16.36 ± 4.72 ^a^	14.07 ± 4.02 ^a^	6.76 ± 1.27 ^a^	3.66 ± 1.31 ^a^	67.27 ± 9.84 ^a^
FBN-80%	2.83 ± 1.47 ^b^	5.00 ± 2.00 ^c^	13.83 ± 3.31 ^b^	17.67 ± 4.46 ^a^	14.83 ± 3.31 ^a^	6.83 ± 1.94 ^a^	3.67 ± 1.37 ^a^	64.67 ± 11.64 ^a,b^
DBN-80%	2.77 ± 1.32 ^b^	5.02 ± 1.66 ^c^	11.22 ± 1.71 ^b^	18.26 ± 3.74 ^a^	11.52 ± 2.93 ^a^	5.65 ± 1.71 ^a^	3.58 ± 1.72 ^a^	58.02 ± 5.41 ^b^

FBN-20/50/80% is fresh buckwheat noodles with 20/50/80% of extruded buckwheat flour; DBN-20/50/80% is dried buckwheat noodles with 20/50/80% of extruded buckwheat flour. The flavour score is in the range of 1 to 5; the colour, appearance, and smoothness score range from 1 to 10; the hardness and stickiness score is in the range of 1 to 20; the elasticity score is in the range of 1 to 25. Detailed meaning for an individual score can be found in Table S1 in the supplementary information. ^a^, ^b^, and ^c^ in a column indicates values are significantly different at *p* < 0.05.

**Table 4:** Transverse relaxation time (*T*_2_) of water in different status/distribution for uncooked FBN and DBN and moisture content for cooked FBN and DBN.

Noodles	*T*_21_/ms	*T*_22_/ms	*T*_23_/ms	Moisture Content/%
FBN-20%	0.21 ± 0.02 ^c^	4.87 ± 0.40 ^b,c^	47.44 ± 13.84 ^c^	65.68 ± 0.10 ^a^
DBN-20%	0.48 ± 0.08 ^b^	6.60 ± 1.78 ^b^	30.30 ± 6.63 ^c^	66.64 ± 0.59 ^a^
FBN-50%	0.19 ± 0.03 ^c,d^	4.87 ± 0.40 ^c^	100.65 ± 14.01 ^a^	65.64 ± 0.37 ^a^
DBN-50%	0.32 ± 0.09 ^b^	7.19 ± 1.60 ^b^	70.00 ± 15.31 ^b^	64.96 ± 0.47 ^a^
FBN-80%	0.16 ± 0.00 ^d^	4.04 ± 0.00 ^d^	127.31 ± 21.37 ^a^	59.03 ± 0.53 ^b^
DBN-80%	0.66 ± 0.09 ^a^	17.34 ± 3.79 ^a^	126.45 ± 9.94 ^a^	66.83 ± 0.04 ^a^

FBN-20/50/80% is fresh buckwheat noodles with 20/50/80% of extruded buckwheat flour; DBN-20/50/80% is dried buckwheat noodles with 20/50/80% of extruded buckwheat flour. *T*_21_, *T*_22_, and *T*_23_ represent the transverse relaxation time of tightly bound water, softly bound water, and free water, respectively. ^a^, ^b^, ^c^, and ^d^ in a column indicate values are significantly different at *p* < 0.05.

**Table 5:** Correlation analysis among different cooking characteristics of FBN and DBN.

Correlation Coefficient		CBR	CL	Hardness/g	Elasticity	Cohesiveness	Chewiness/g	Resilience
*T*_21_	FBN	−0.938	−0.916	0.998 *	−0.826	−0.997 *	0.987	0.826
	DBN	0.881	0.682	−0.572	0.349	0.373	−0.996	−0.92
*T*_22_	FBN	−0.998 *	−0.681	0.888	−0.982	−0.945	0.840	0.982
	DBN	0.999 *	0.961	−0.91	0.779	0.795	−0.812	−0.59
*T*_23_	FBN	0.974	0.858	−0.981	0.890	0.999 *	−0.958	−0.89
	DBN	0.914	0.996	−0.999 *	0.954	0.962	−0.542	−0.254
*A*_21_	FBN	0.986	0.827	−0.968	0.915	0.994	−0.940	−0.915
	DBN	−0.787	−0.943	0.981	−0.998 *	−1.000 *	0.321	0.010
*A*_22_	FBN	−0.951	−0.900	0.994	−0.847	−0.999 *	0.980	0.847
	DBN	0.997	0.970	−0.925	0.802	0.817	−0.789	−0.559
*A*_23_	FBN	0.716	1.000 **	−0.939	0.525	0.880	−0.968	−0.525
	DBN	0.791	0.945	−0.982	0.998 *	0.999 *	−0.326	−0.016
MC	FBN	−0.999 *	−0.684	0.891	−0.981	−0.947	0.843	0.981
	DBN	0.574	0.281	−0.141	−0.111	−0.085	−0.929	−0.998 *

* *T*_21_, *T*_22_, and *T*_23_ represent the transverse relaxation time of tightly bound water (TBW), softly bound water (SBW) and free water (FW), respectively. ** *A*_21_, *A*_22_, and *A*_23_ represent the proportion of tightly bound water (TBW), softly bound water (SBW) and free water (FW), respectively. CBR, cooking breakage ratio; CL, cooking loss; MC, moisture content.

## Data Availability

All data generated or analysed during this study are included in this article.

## Supplementary Materials

The following are available online at https://www.mdpi.com/2304-8158/10/1/187/s1: Figure S1: CPMG and IR signal of FBN-20% and DBN-20%; Table S1: Sensory parameters and standard of the score for buckwheat noodles; Table S2: Relative amount of water in different states/distribution for uncooked FBN and DBN (A21 for tightly bound water (TBW); A22 for softly bound water (SBW); A23 for free water (FW)).

## Abbreviations

FBN	fresh buckwheat noodles
DBN	dried buckwheat noodles
TBW	tightly bound water
SBW	Softly bound water
FW	free water
*T*_21_	the transverse relaxation time of tightly bound water
*T*_22_	softly bound water
*T*_23_	free water
A_21_	the proportion of tightly bound water
*A*_22_	softly bound water
*A*_23_	free water
OCT	optimal cooking time
CL	cooking loss
CBR	cooking breakage ratio
MC	moisture content
