# Supplementary material for: Quality Differences between Fresh and Dried Buckwheat Noodles Associated with Water Status and Inner Structure
Source: Foods. 2021 Jan 18;10(1):187. doi: 10.3390/foods10010187 (PMC7831939; doi:10.3390/foods10010187)
Supplement: Supplementary file 1 [file foods-10-00187-s001.pdf]

Supplementary Materials

Table S1: Sensory parameters and standard of the score for buckwheat noodles

Table S2: Relative amount of water in different states/distribution for uncooked FBN and DBN (A21 for tightly bound water (TBW); A22 for softly bound water (SBW); A23 for free water (FW))

Figure S1: CPMG and IR signal of FBN-20% and DBN-20%

Table S1. Sensory parameters and standard of the score for buckwheat noodles.

Sensory parameters (Full score)	Score			
**Colour (10)**	**Light colour and bright 8-10**	Brown and less bright 5-7	Dark-brown 1-4	-
**Appearance (10)**	Smooth and irregular shape 8-10	Less smooth and in shape 5-7	Rough and unshaped 1-4	-
**Hardness (20)**	Suitable hardness 17-20	Harder or softer 12-16	Over hard or soft1-12	-
**Elasticity (25)**	Elastic 21-25	Poor elastic 16-20	Worse elasticity 11-15	Worst elasticity 1-10
**Stickiness (20)**	Non-sticky 16-20	Less sticky 11-25	Sticky 6-10	Very sticky 1-5
**Smoothness (10)**	Smooth 8-10	Less smooth 5-7	Coarse 1-4	-
**Flavour (5)**	Buckwheat flavour 5	Non-smell 3-4	Smell 1-2	-

Table S2. Relative amount of water in different states/distribution for uncooked FBN and DBN (A_{21} for tightly bound water (TBW); A_{22} for softly bound water (SBW); A_{23} for free water (FW)).

Noodles	A_{21}/%	A_{22}/%	A_{23}/%
FBN-20%	$18.03 \pm 0.29^{b1\ B}$	$81.60 \pm 0.24^{a1\ A}$	$0.37 \pm 0.28^{b1\ B}$
DBN-20%	$93.58 \pm 0.67^{a2\ A}$	$3.64 \pm 0.90^{a2\ B}$	$2.78 \pm 0.25^{c2\ A}$
FBN-50%	$19.63 \pm 1.32^{b1\ B}$	$78.27 \pm 1.55^{b1\ A}$	$2.10 \pm 0.24^{a1\ B}$
DBN-50%	$89.86 \pm 0.77^{b2\ A}$	$3.66 \pm 0.67^{a2\ B}$	$6.34 \pm 0.23^{b2\ A}$
FBN-80%	$24.88 \pm 0.12^{a1\ B}$	$72.51 \pm 0.14^{c1\ A}$	$2.61 \pm 0.07^{a1\ B}$
DBN-80%	$87.65 \pm 1.43^{c2\ A}$	$3.85 \pm 1.12^{a2\ B}$	$8.50 \pm 1.59^{a2\ A}$

*The a1, b1 and c1 in a column indicate the proportion of TBW, SBW, and FW respectively within FBN-20/50/80 % is significantly different at $p<0.05$; The a2, b2, and c2 in a column indicate the proportion of TBW, SBW, and FW respectively within DBN-20/50/80 % is significantly different at $p<0.05$; The A and B of FBN and DBN with the same addition of extruded buckwheat flour indicate significantly different at $p<0.05$.

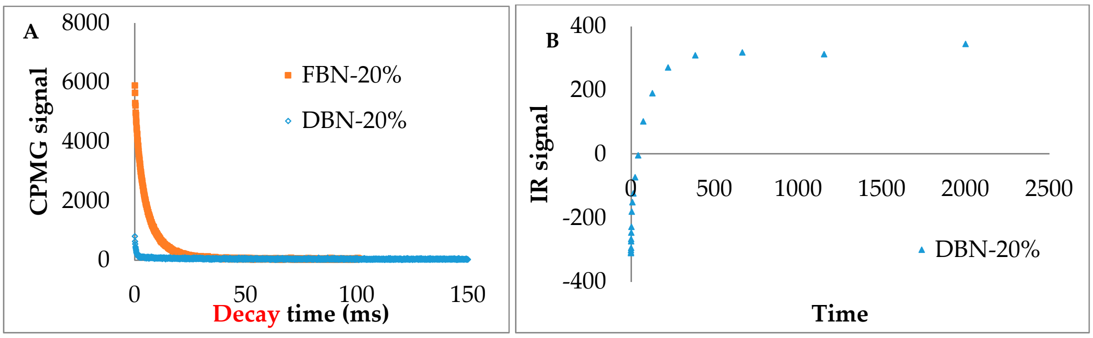

Figure S1. CPMG (A) and IR (B) signal of noodles

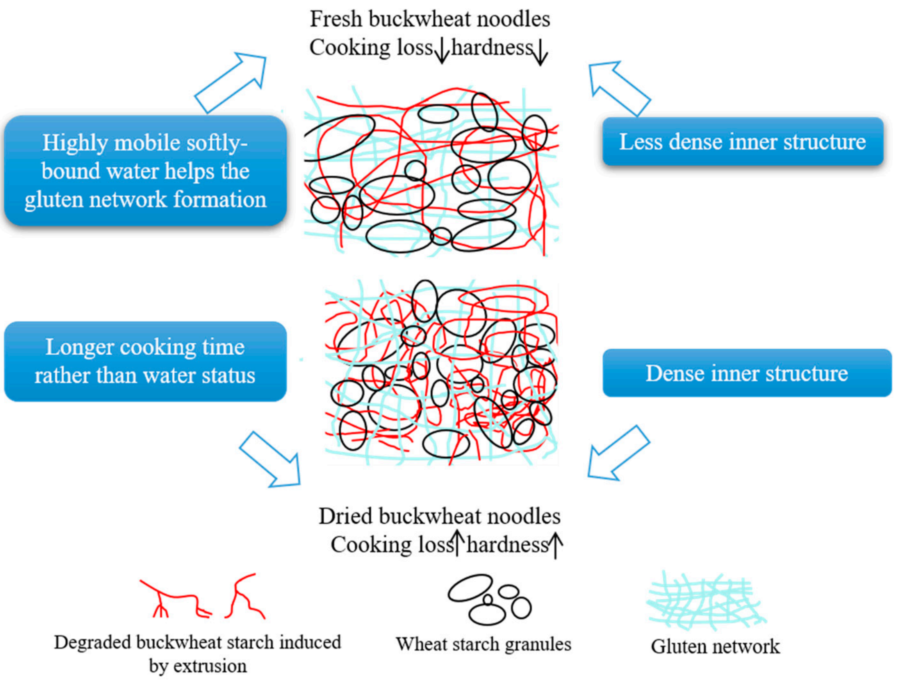

Figure S2. Graphical abstract
